# COVID-19 in hematological malignancy patients: A protocol for a systematic review and meta-analysis

**DOI:** 10.1097/MD.0000000000021376

**Published:** 2020-08-28

**Authors:** Can Chen, Qianping Weng, Yiwei Li, Pengfei Shi, Shenxian Qian

**Affiliations:** a Department of Hematology; b Department of Intensive Care Unit, Affiliated Hangzhou First People's Hospital, Zhejiang University School of Medicine, Hangzhou, Zhejiang, PR China.

**Keywords:** COVID-19, hematological malignancy, meta-analysis, protocol, systematic review

## Abstract

**Background:**

COVID-19 is an international outbreak of the respiratory illness caused by severe acute respiratory syndrome coronavirus 2 (SARS-CoV-2). The diseases themselves, as well as the intensity of chemotherapy, lead to significant immunosuppression, leading hematological malignancy patients susceptible to infections.

**Methods:**

This protocol will be performed according to the Meta-analysis of Observational Studies in Epidemiology (MOOSE) guidelines and reported follow the Cochrane Collaboration Handbook and the Preferred Reporting Items for Systematic Reviews and Meta-Analysis (PRISMA) guidelines. Electronic databases of PubMed, MEDLINE, Google Scholar, Web of science, Cochrane Library, EMBASE, CNKI, CMB, and Wangfang database from the inception to present will be comprehensively and systematically searched without limitations of language, date, and publication status. Observational, retrospective cohort, prospective case-control, cohort studies, cross-sectional studies, or clinical trials will be included. All assessment of study selection, data extraction, and study quality assessment will be independently performed by 2 reviewers. RevMan V.5.3 program and Stata V.12.0 software will be utilized for the methodological quality assessment and statistical analysis.

**Results:**

The result of this systematic review will provide evidence for clinicians on the management of COVID-19 patients with hematological malignancy.

**Conclusion:**

This systematic review will help raise awareness and guide management of COVID-19 patients with hematological malignancy, as well as to improve outcomes in this population.

**Ethic and dissemination:**

The content of this article does not involve moral approval or ethical review because no individual data will be collected.

**PROSPERO registration:**

CRD42020187493.

## 1 Introduction

In December 2019, a novel type of coronavirus was reported in Wuhan and named COVID-19. It is an international outbreak of the respiratory illness caused by severe acute respiratory syndrome coronavirus 2 (SARS-CoV-2).^[1]^ Previous studies reported that most of patients (81%) with COVID-19 presented mild illness, 14% had severe illness requiring hospitalization and supplemental oxygen, and the remaining 5% presented severe illness might result in death due to massive alveolar damage and progressive respiratory failure which need critical care.^[1,2]^ As of May 26, a total of 549,5061 cases have been confirmed and 34,6232 deaths have been reported across 188 countries or regions.^[3]^

Currently, COVID-19 is spreading rapidly through Europe and North America.^[4]^ Until now, there is no effective vaccine or specific antiviral therapy. Containment and rigorous case findings are the only measures to prevent or delay community spread.^[5]^ Immunosuppressed patients usually susceptibility to infection with SARS-CoV-2, especially for cancer patient. Of these, due to the intensity of chemotherapy and the disease itself, hematological malignancy patients are at increased risk of infection.^[6,7]^

Knowledge of the COVID-19 disease process in patients hematologic malignancies is rare. Previous publications reported that patients with cancer was approximate 1% among COVID-19 patients.^[6–9]^ Initial reports from China suggested that patients with cancer had a higher risk of severe complications of respiratory viruses, such as advanced age and comorbidities.^[1,9]^ Compared to patients without cancer, severe events (ICU admission, invasive ventilation or death) were higher in cancer patients (39% vs 8%).^[1]^ Also, the symptom of patients with cancer may develop more rapidly than others. Meanwhile, anticancer therapy, including chemotherapy, radiotherapy, targeted therapy, or immunotherapy will aggravate the disease progression, make treatment difficult.^[10]^

Due to the specific disease biology or associated therapy, different hematological malignancies may also be associated with particular COVID-19-associated risks.^[11]^ Leukemia patients with COVID-19 are under immunocompromised which may lead to superimposed bacterial or fungal pneumonia. Result from impaired humoral response caused by disease- or treatment-related hypogammaglobulinemia, patients with lymphoid malignancies are also at higher risk of infection. Because hematological malignancy patients often present with a cough with or without fever, after a negative SARS-CoV-2 test, symptoms are likely to be neglected.^[12,13]^ The diagnosis is often missed and delayed, leading to worse outcome. Most patients will suffer from postponed chemotherapy or hematopoietic stem cell transplant (HSCT) result from a shortage of isolation beds and blood products. For patients with low risk, such delay of chemotherapy initiation may negatively affect prognosis.^[1]^ Since blood products shortage has already begun in most affected countries, hematological malignancy patients will also get adversely affected from it.^[14]^ There are limited studies with hematological malignancies and the ramifications in that specific population are not well known. We tried to gather evidence to systematically review the clinical features, treatments, prognosis of COVID-19 patients with hematological malignancy.

## 2 Methods

### 2.1 Study registration.

This protocol is guided by the Preferred Reporting Items for Systematic Reviews and Meta-Analysis Protocol (PRISMA-P) statement and performed according to the Meta-analysis of Observational Studies in Epidemiology (MOOSE) guidelines.^[15]^ We will use RevMan 5.3 software to perform meta-analysis and follow the Cochrane Collaboration Handbook and the PRISMA statement guidelines to report its result.^[16]^ This protocol was registered on PROPERO (Registration number: CRD42020187493) on 22 May 2020.

### 2.2 Eligibility criteria

#### 2.2.1 Types of participants

Patients aged 18 years and older who have diagnosed with hematological malignancy infected with COVID-19 pneumonia with a positive lab test of dual fluorescence polymerase chain reaction (PCR) or oropharyngeal swab specimen, or with clinical diagnosis cases according to clinical guidelines. No restrictions will be applied in terms of sex, race, disease duration, or disease severity.

#### 2.2.2 Types of studies

Observational, retrospective cohort, prospective case-control, cohort studies, cross-sectional studies, or clinical trials will be eligible for inclusion. No language, date, or publication status restrictions will be applied.

#### 2.2.3 Types of outcome measures

Our primary outcomes will include survival and death (mortality, all-cause mortality, progression free survival (PFS) and overall survival (OS). Secondary outcomes will include overall response, progressive disease, relapse, proportion of patients with adverse events (acute respiratory distress, respiratory failure, sepsis, shock, cytokine release syndrome, multiple organ dysfunction syndrome, etc.), proportion of patients required mechanical airway protection or hemodynamic support, lab characteristics (routine blood test, lymphocyte subgroups, lactic dehydrogenase, D-dimer, aPTT, etc.), proportion of patients admitted to ICU, length of stay in hospital. If other outcomes are reported in the eligible studies, these will also be extracted.

#### 2.2.4 Exclusion criteria

The exclusion criteria are as follows:

1. Studies with overlapping data.
2. Conference abstracts, case reports, series, abstract-only articles, letters to the editor, editorials, and expert opinions.
3. Studies that enrolled less than 10 patients.
4. Missing or insufficient data that cannot be obtained after contacting original authors.

### 2.3 Data sources and search strategy.

#### 2.3.1 Electronic searches

An electronic search will be performed in the following databases: PubMed, MEDLINE, Google Scholar, Web of Science, Cochrane Library, EMBASE, CNKI, CMB, and Wangfang database from the inception to June 2020. The proposed database search using the MeSH search terms have been shown in Table 1. Reference lists will be collected in Endnote software 8.1 and duplicate articles will be deleted. The initial selection will be based on the screening of titles and abstracts of the retrieved articles to exclude obvious irrelevant studies. Potentially relevant studies will be retrieved and evaluated in full-text versions separately by 2 reviewers. If authors are similar or data are extracted from the same database, the study period will be noted. Only the latest study will be included if the study period overlaps. If consensus is not reached, a third reviewer will play an arbiter role.

**Table 1:**
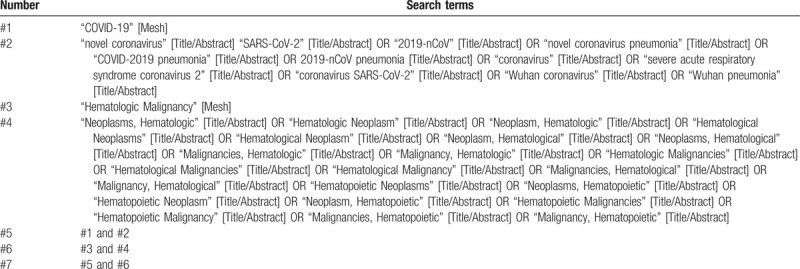
Search strategy applied in PubMed database.

#### 2.3.2 Other resources

**Figure 1:**
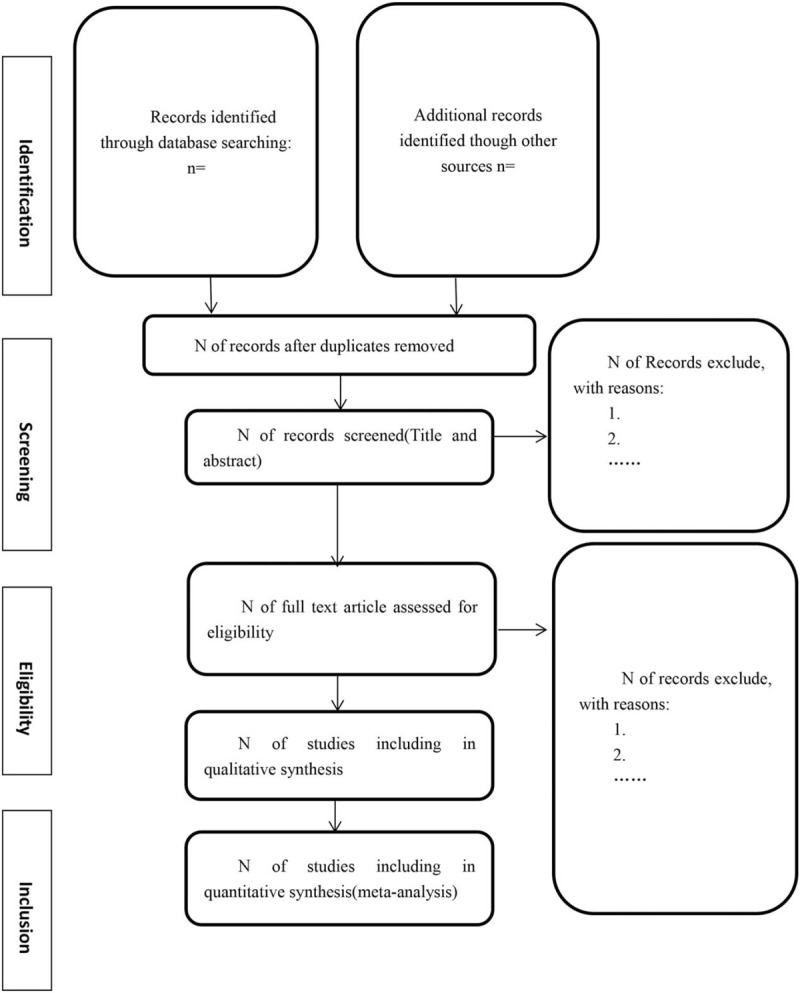
Flow diagram of literature search.

Additional relevant articles will be identified by scanning the reference lists of relevant original articles. We will also seek the COVID-19 Study Registry (https://covid-19.cochrane.org/) and COVID-evidence (https://covid-evidence.org/). The process of study selection is illustrated following a PRISMA guidelines (Fig. 1).

### 2.4 Data collection and analysis

#### 2.4.1 Data extraction and management

The data extraction will be performed by 2 independent reviewers using a standardized data extraction sheet. The data extracted will include information on study data, characteristic of participants, interventions, outcomes of patients and any outcomes of significance to the analysis, as well as risk estimate confounders. We will contact primary authors to request for any data missing or clarifications needed. To ensure the accuracy and consistency of the extracted data, cross-checked will be used by 2 reviewers. Any disagreements will be judged by a third reviewer. The following data will be extracted from the eligible studies:

1. Study data: author details, year of publication, country, sample size, study design, follow up period, publication status.
2. Characteristic of participants: mean age, sex, baseline situation, hematological malignancy diagnosis, severity of COVID-19 pneumonia, ECOG performance status, comorbidity.
3. Interventions: Chemotherapy, immunotherapy, supportive care and HSCT, and therapeutic strategy (such as type, dosage, frequency, duration, etc.).
4. Outcomes: mortality, all-cause mortality, PFS, OS, proportion of patients with progressive or relapsed disease, or required mechanical airway protection or hemodynamic support, the rates and types of adverse events.

#### 2.4.2 Risk of bias assessment

The quality of included studies will be independently assessed by 2 reviewers, any discrepancy will be resolved through discussion or judged by a third reviewer. The standard checklists will be used as followed:

1. The quality of studies, including case-control studies and cohort studies, will be assessed using the Newcastle-Ottawa Scale (NOS) independently by 2 reviewers, which consisted of 3 parameters: selection, comparability, and exposure assessment. Studies with a total NOS score of 5 or more are considered to be of moderate to high quality, whereas those with an NOS score of less than 5 are considered low-quality.
2. The assessment of cross-sectional studies by using criteria adapted from the Agency for Healthcare Research and Quality, which including 11 items.^[17]^ Each item will be scored as “Yes”, “No” and “Unclear” depending on whether each study met these criteria or not. “Unclear” record will be made if there is insufficient detailed in the publication.
3. Cochrane Handbook for systematic reviews of interventions will be used for clinical trials, which covers: sequence generation, allocation concealment, blinding of participants and personnel, blinding of outcome assessments, incomplete outcome reporting, selective reporting, and other sources of bias. Each of the domains will be scored as “low risk”, “high risk” or “Unclear”.^[18]^

#### 2.4.3 Data synthesis and heterogeneity assessment

All included studies will be reviewed and assessed by 2 steps. First, we will collect and categorized the relevant data sources after data extraction. Then the extracted data will be analyzed using RevMan V.5.3 program and Stata V.12.0 software to perform the meta-analysis. Dichotomous outcomes will be determined by using risk ratios (RRs) with a confidence interval (CI) of 95%. For continuous variables will be recorded as the mean differences (MD) with 95% CI.

To determine the impact of the statistical heterogeneity on the meta-analysis, we will primarily use forest plots to assess any sign of potential heterogeneity visually. Then Cochrans Q and Higgins *I*^2^ statistic will be used to assess the presence of statistical heterogeneity and quantified the heterogeneity between studies. If a *P* value of >.10 for the Chi^2^statistic or an *I*^2^ < 50%, heterogeneity will be considered as low, and the fixed-effect model will be chosen; Otherwise, the random-effects model will be applied due to the severe heterogeneity is observed. Where substantial heterogeneity will be confirmed, subgroup analysis and sensitivity analysis will be performed to investigate the possible sources of heterogeneity. However, if heterogeneity is considerable high, the meta-analysis should be avoided, and narrative comprehensive synthesis will be considered.

#### 2.4.4 Subgroup analysis

Subgroup analysis will be designed based on the characteristics which may be causes of heterogeneity observed in the primary analysis. Before performing the analysis, we will confirm the number of studies is adequate. The tentative Subgroups for analysis are as follows:

1. Population characteristics.
2. Study design and research quality.
3. Sample size, location, and ethnicity.
4. Follow-up period.

#### 2.4.5 Sensitivity analysis

We will conduct sensitivity analyses to identity the reliability and stability of individual studies in the overall effect estimate by removing one by one from the analysis.

#### 2.4.6 Reporting bias analysis

Reporting bias will be carried out using a funnel plot and Egger regression test if a sufficient number of articles are included. Asymmetry of the funnel plot or a *P* value of Egger regression test less than .05 will indicate the high risk of publication bias.^[19]^

#### 2.4.7 Evidence evaluation

The strength of evidence will be assessed on the Grading of recommendations, assessment, development, and evaluation (GRADE) system. It consists 5 evaluation levels: limitations, indirectness, inconsistency (heterogeneity), imprecision, and/or publication bias and quality of evidence for each outcome will be graded as high, moderate, low, or very low.^[20]^

## 3 Discussion

Immunocompromised host especially hematological malignancy patients are susceptible to infected COVID-19 and may have inferior outcome. This systematic review will be divided into 5 sections: identification, study inclusion, data extraction, data synthesis, and study quality assessment. We hope that the result would help the physicians to choose a suitable chemotherapy timing and intensity. We believe that our result will provide important information for the epidemiological data, clinical characteristics, treatment, and outcome of COVID-19 process in patients with hematologic malignancies.

## Author contributions

**Conceptualization:** Pengfei Shi, Shenxian Qian.

**Data curation:** Can Chen, Yiwei Li.

**Formal analysis:** Can Chen, Qianping Weng.

**Funding acquisition:** Shenxian Qian.

**Methodology:** Can Chen, Qianping Weng.

**Project administration:** Can Chen, Pengfei Shi.

**Software:** Yiwei Li, Pengfei Shi.

**Supervision:** Pengfei Shi, Shenxian Qian.

**Validation:** Can Chen, Yiwei Li, Qianping Weng, Shenxian Qian, Pengfei Shi.

**Writing – original draft:** Can Chen, Yiwei Li.

**Writing – review & editing:** Can Chen, Yiwei Li, Qianping Weng, Shenxian Qian, Pengfei Shi.
